# 296 A Pilot Study of Hand Autografts Treated with Autologous Skin Cell Suspension

**DOI:** 10.1093/jbcr/irad045.271

**Published:** 2023-08-29

**Authors:** Paige Deville, Jeffrey E Carter, Madeline Lechtenberg, Herbert A Phelan

**Affiliations:** LSUHSC New Orleans Department of General Surgery, New Orleans, Louisiana; University Medical Center New Orleans & LSUHSC, River Ridge, Louisiana; LSUHSC Medical School New Orelans, New Orleans, Louisiana; LSUHSC New Orleans Department of General Surgery, New Orleans, Louisiana

## Abstract

**Introduction:**

Our group has previously studied the use of autologous skin cell suspensions in conjunction with meshed autografts for the treatment of hand burns. These studies lacked scar assessments and long-term follow up, however. The aim of this study was to compare the point estimates for prospectively collected scar characteristics in hand burns treated with meshed autograft and an ASCS solution vs split thickness autograft alone (STAG). As these point estimates are unknown, this study was considered to be hypothesis-generating to aid in future power calculations.

**Methods:**

A retrospective review was conducted to identify all subjects operated on for deep partial or full thickness hand burns at our ABA-verified burn center between April 2018 to April 2022. Eligible subjects were attempted to be contacted by phone and offered study participation. No financial enticements were offered beyond validating parking. All consenting subjects underwent measurement of scar characteristics by the Patient-Observer Scar Assessment Score (POSAS) by one of two dedicated study personnel. Two cohorts were created: those hands treated with 2:1 meshed autograft plus application of an ASCS solution (MA/ASCS) versus those hands treated with split thickness sheet, piecrust, or 1:1 meshed autograft alone (STAG). Values are reported as median and interquartile range.

**Results:**

90 subjects fit the study criteria overall, with 20 able to be contacted (22%) and 17 of those consenting to participate (18%). The MA/ASCS group (n=8) were seen at 8 [3.75,17.25] months after injury while the STAG group (n=9) presented at 15 [9,21] months from injury. There was no significant difference in age between the two cohorts (35.5 years [29.75, 55.5] vs 51 years [45, 53], p=0.53), but the MA/ASCS group had significantly larger %TBSA burns overall (20% [5%, 10%] vs 2% [1%, 4%], p=0.01). Scar assessments were found to be comparable between the two groups as the POSAS-patient scores were 34.5 [13.5, 51] in MA/ASCS and 37 [14, 40] in STAG, while the POSAS-observer scores were 22 [20.25, 29] in the MA/STAG group and 21 [9, 25] in the STAG group. For a noninferiority trial of ASCS with POSAS-observer as the primary endpoint, an alpha of 0.05, 80% power, and a conservative noninferiority margin M of 10%, a randomized clinical trial would need 184 subjects.

**Conclusions:**

The point estimates for scar assessment of hand burns treated with ASCS and 2:1 meshed autograft are so close to STAG alone, the resources necessary to perform a definitive RCT would require a degree of resources that are probably prohibitive. Alternative endpoints should be considered in judging the performance of ASCS in hand burns, such as the amount of donor skin needed to close the wound.

**Applicability of Research to Practice:**

This study can provide important information in changing clinical practice for the treatment of hand burns.

